# IQ, Educational Attainment, Memory and Plasma Lipids: Associations with Apolipoprotein E Genotype in 5995 Children

**DOI:** 10.1016/j.biopsych.2010.10.033

**Published:** 2011-07-15

**Authors:** Amy E. Taylor, Philip A.I. Guthrie, George Davey Smith, Jean Golding, Naveed Sattar, Aroon D. Hingorani, John E. Deanfield, Ian N.M. Day

**Affiliations:** a Bristol Genetic Epidemiology Laboratories, University of Bristol, Bristol, United Kingdom; b MRC Centre for Causal Analyses in Translational Epidemiology, University of Bristol, Bristol, United Kingdom; c Centre for Child and Adolescent Health, University of Bristol, Bristol, United Kingdom; d School of Social and Community Medicine, University of Bristol, Bristol, United Kingdom; e BHF Glasgow Cardiovascular Research Centre, University of Glasgow, Glasgow, United Kingdom; f Department of Epidemiology and Public Health, Institute of Child Health, University College, London, United Kingdom; g Vascular Physiology Unit, Institute of Child Health, University College, London, United Kingdom

**Keywords:** APOE, children, cognitive function, IQ, lipids, memory

## Abstract

**Background:**

Apolipoprotein E (*APOE*) genotype (ε2/ε3/ε4: rs429358 ε4 allele; rs7412 ε2 allele) is strongly associated with both lipid levels and Alzheimer's disease. Although there is also evidence of milder cognitive impairment in later life in carriers of the *APOE* ε4 allele, there have been few studies investigating the impact of *APOE* genotype on cognitive function in children.

**Methods:**

We determined *APOE* genotype in 5995 children from the Avon Longitudinal Study of Parents and Children and investigated associations between *APOE* genotype and plasma lipids (at age 9), IQ (at age 8), memory (at ages 8 and 10), and performance in school attainment tests (at ages 7, 11, and 14).

**Results:**

Observed genotype group counts were consistent with Hardy–Weinberg equilibrium (*χ*^2^*p* value = .84). There were strong relationships between *APOE* genotype and low-density lipoprotein cholesterol, high-density lipoprotein cholesterol, and triglycerides, which follow the same patterns as in adults. There was no strong evidence to suggest that *APOE* genotype was associated with IQ (all *p* values ≥ .46), memory function (*p* ≥ .35), or school attainment test results (*p* ≥ .28).

**Conclusion:**

Although *APOE* genotype does have strong associations with lipid levels in childhood, there does not seem to be meaningful effects on cognitive performance, suggesting that any detrimental effects of the ε4 allele on cognitive function are not important until later life.

There are two well-known coding polymorphisms of apolipoprotein E (APOE), resulting in arginine > cysteine amino acid changes at positions 130 (rs429358) and 176 (rs7412). These changes determine alleles ε2/ε3/ε4, having frequencies of 8%, 78%, and 14%, respectively, in the UK population (1). The *APOE* ε4 allele represents a major susceptibility factor for late onset Alzheimer's disease (LOAD), with carriers having approximately a threefold risk (over tenfold for homozygotes) of developing LOAD compared with noncarriers (2,3). The mechanism linking *APOE* genotype and LOAD remains obscure, although isoform-dependent effects on β amyloid deposition and clearance, synaptic signaling, and inflammatory responses have been reported (4,5).

The *APOE* ε4 allele might also be associated with domain-specific cognitive decline in normal aging (6–14). Memory decline in particular has been noted in several studies, including one study of almost 6000 elderly adults (15), as being more marked in ε4 carriers (12,15–17). A recent model predicted that long-term memory decline in ε4 carriers began at age 50–60 years compared with age 70–80 years in noncarriers (12). Beginning at approximately age 45, persons who carry the *APOE* ε4 allele demonstrate an accelerated evolution of regional brain LOAD changes according to Braak criteria (18). However, the effects of the *APOE* genotype on cognitive functioning in adulthood are inconsistent. Jorm *et al*. (19) found no strong evidence for an effect of ε4 allele carriage on cognitive function in 6560 adults ranging in age from 20 to 64 years, and in a meta-analysis of data from 38 studies of participants ages 45 years and above, ε4 carriers exhibited minor reductions in global cognitive functioning, episodic memory, and executive functioning. However, the observed differences were <.1 SD units (20).

Given that the domains of cognitive function that have demonstrated association with *APOE* genotype are similar to the early signs of LOAD and the timing of the onset of this cognitive decline, it has been suggested that ε4-related cognitive decline might be caused by preclinical LOAD (13). However, the three common isoforms of APOE have major effects on lipoprotein fractions and subsequent risk of cardiovascular disease (21) as well as drug prescribing (22). Individuals with the ε4 allele tend to have higher levels of total and low-density lipoprotein cholesterol (LDLc) and lower levels of high-density lipoprotein cholesterol (HDLc) than noncarriers. For triglycerides a U-shaped distribution is observed, with ε3 homozygotes having the lowest levels and ε4 and ε2 heterozygotes having intermediate levels and ε4 and ε2 homozygotes having the highest levels. Thus, it is possible that associations between *APOE* genotype and cognitive function outside of LOAD might also have vascular origins. The *APOE* ε4 allele has been shown to be a risk factor for ischemic cerebrovascular disease (23).

The influence of *APOE* alleles on cognition at an early age would be of particular interest, because it would potentially aid mechanistic understanding. There have been relatively few studies of *APOE* in relation to cognition in children; most of these have been small and therefore of limited power to detect differences between individual genotype groups. Deary *et al*. (24), in a retrospective study of 173 participants in the Lothian study, found no difference in performance by ε4 carrier status in the Moray House IQ test at age 11 years, and Turic *et al*. (25) similarly found no differences in *APOE* genotype distribution in 101 high-IQ and 101 average-IQ children. In a study of 109 11–16-year-olds in California, Bloss *et al*. (26) found some evidence that cognitive function was lower only in ε4 carriers who also had a family history of Alzheimer's disease. However, in children, total spatiotemporal wave activity patterns have been shown to differ between ε3 homozygotes and ε4 carriers (27). In addition, a magnetic resonance imaging study of 239 healthy children showed that ε4 carriers had lower cortical thickness in the left entorhinal region of the brain compared with noncarriers (27). There is also evidence that the ε4 allele might actually be protective for brain development and cognitive function in early childhood. Infants carrying the ε4 allele performed better in mental development tests in a study in Mexico, and amongst children in Brazil, who had had heavy diarrhea burdens in their first 2 years of life, ε4 carriers showed better verbal fluency than noncarriers (28,29). This has led some to hypothesize that ε4 might be an example of antagonistic pleiotropy, with the ε4 allele being beneficial in early life but detrimental later in life (29,30). This could be due to the higher cholesterol levels seen in ε4 carriers, because cholesterol is essential for neurodevelopment (31).

The purpose of this study was to obtain more precise estimates of the effects of all six *APOE* genotypes on cognitive performance in childhood, in the context of the prevailing childhood blood lipid profile. We analyzed *APOE* genotype in relation to IQ at age 8, memory tasks at ages 8 and 10, and school age educational performance tests (at ages 7, 11, and 14) in children from the ALSPAC study (Avon Longitudinal Study of Parents and Children). In parallel, we analyzed the *APOE* genotype in relation to plasma lipid profiles.

## Methods and Materials

### Study Population

The ALSPAC study (http://www.bristol.ac.uk/alspac) is a prospective study, established to explore child health and development (32). The initial ALSPAC sample consisted of 14,541 pregnant women from Bristol, United Kingdom, with expected delivery dates between April 1991 and December 1992, resulting in 14,062 live births. At age 7, a further 548 eligible children were added to the sample, making a total sample size of 14,610 for analyses. Detailed information on the children has been collected via questionnaires since birth and at annual clinic visits since the age of 7.

### Genotyping

#### DNA Samples

Deoxyribonucleic acid samples were available for this study for 7091 children (63% of the 11,343 ALSPAC children with DNA samples available). Genotyping of the three main allelic variants (ε3, ε4, and ε2) of *APOE* was undertaken by integrated single-label liquid phase assay. Full details of the method have been published previously (1). Polymerase chain reaction products were analyzed with a 384-well LightTyper instrument (Roche Diagnostics, GmbH, Indianapolis, Indiana), and genotypes were determined with Light-Typer software, Ver. 1. Duplicate DNA samples (identities unknown during genotyping) were analyzed to validate the assay, and a random sample (of 100 wells) was called independently by two investigators as a validation of genotype calling.

#### Lipids

Nonfasting blood samples were taken during clinic visit at age 9 (age range 8.8–11.7 years). Plasma lipids (total cholesterol, triglycerides, and HDLc) were measured by modification of the standard Lipid Research Clinics Protocol with enzymatic reagents for lipid determinations (33). The LDLc was estimated with the Friedewald equation (34).

### Cognitive Function Measures

#### Clinic Assessments

The IQ was measured at a clinic held when the children were 8.5 years of age (mean: 8.7 years; range: 7.5–9.4 years) with the Wechsler Intelligence Scale for Children (35). A shorter version of the scale was used in which alternate items (always starting with Item 1 on the standard form) were used for 9 of 10 subtests. Scores from the verbal and performance subscales were used as outcome measures as well as overall IQ score. A measure of speech and language, the Wechsler Objective Language Dimensions (WOLD) test (36) was administered at the same clinic. Reading level was assessed at clinic at age 7.5 years (mean: 7.5 years; range: 6.9–8.0 years) with measures based on the Wechsler Objective Reading Dimensions (WORD) test (37).

Short-term memory at age 8 was measured in clinic with an adaptation of the Nonword Repetition Test (38). Children were asked to repeat 12 nonsense words of three, four, and five syllables after hearing them on an audio cassette. The outcome measure is the number of words repeated correctly. Working memory at age 10 was assessed in clinic via the Counting Span Working Memory Task (39). This test involved counting and recalling numbers of dots on screens, which were administered in sets of two, three, four, and five screens. Two scores were recorded from this test; the span score represents the number of correctly recalled sets, with a maximum score of 5 in increments of .5, and the global score represents the total number of screens correct, with a maximum of 42 (40).

#### School Assessments

In addition to clinic measures of cognitive function, we used the results of nationally administered school-based tests (SATS) (for more detailed information see website: http://curriculum.qcda.gov.uk/). These are undertaken in Year 2 (key stage 1, age 6–7 years), Year 6 (key stage 2, age 10–11 years), and Year 9 (key stage 3, age 13–14 years). For key stage 1, English (reading and writing) and math scores were categorized into three groups: below average (W, 1), average (2a, 2b, 2c), and above average (3+) on the basis of the expected attainment for this age group. For key stages 2 and 3, total scores obtained in the tests for the three core subjects (English, math, and science) were used as the outcome measure.

### Statistical Analysis

Individuals of known nonwhite ethnic origin (*n* = 547) were excluded from all analyses. Where siblings and multiple births were present, the first-born in the study was kept, and the others (*n* = 172) were dropped from all analyses.

Hardy–Weinberg equilibrium tests were performed on the entire sample of genotyped children (excluding siblings and those of nonwhite ethnicity) and on the samples with available lipid or cognitive function measures to assess the possibility of sampling bias due to nonattendance at clinic.

Means and SDs of total cholesterol, LDLc, and HDLc levels were calculated for each genotype. Geometric means and interquartile ranges are presented for triglycerides, due to skewed distributions.

Age- (in months) and gender-adjusted associations between genotypes and total cholesterol; LDLc; HDLc; triglycerides (log-transformed); total, verbal, and performance IQ; WORD and WOLD scores; memory tests; and key stage 2 and 3 test scores were assessed by linear regression. In all regression models, each *APOE* genotype was considered separately with ε3 homozygotes as the reference group. Heterogeneity of associations of *APOE* genotypes with each outcome were assessed by analysis of covariance models and *p* values reported. Chi-square tests were used to look at associations between *APOE* genotypes and key stage 1 test results. Direct associations between plasma lipids (as continuous variables and in quartiles) and cognitive function measures were assessed by linear regression, adjusted for age, gender, and maternal education and household socioeconomic status.

### Ethical Approval

Ethical approval was obtained from the ALSPAC Law and Ethics Committee and local research ethics committees. Parental consent and assent of the child were obtained for all measurements made.

## Results

In total, 95% of samples were successfully genotyped. The number of children with genotype information available for the analyses was 5995, after excluding children of known nonwhite ethnicity and siblings. Lipid measures on a subset of 2875 of those genotyped and IQ measures for a subset of 3925 of those genotyped were available. There was no strong evidence of a gender difference in genotype distribution (*p* = .16) or of deviation from Hardy–Weinberg equilibrium for the whole sample (*χ*^2^ = 1.42, *p* = .84) or when the sample was restricted to children with genotype and lipid data (*n* = 2875, *χ*^2^ = 4.53, *p* = .34) or genotype and IQ data (*n* = 3925, *χ*^2^ = 1.97, *p* = .74).

### Lipids

Levels of total cholesterol and LDLc increased according to the number of ε4 alleles and decreased according to the number of ε2 alleles (*p* < .0001) (Table 1). Conversely, levels of HDLc decreased according to the number of ε4 alleles and increased according to the number of ε2 alleles (*p* < .0001). A U-shaped curve was found for triglycerides where levels increased according to the number of ε2 and ε4 alleles. Tables S1 (male subjects) and S2 (female subjects) in Supplement 1 describe lipid levels in relation to *APOE* genotype for boys and girls. Girls demonstrated higher levels of total, LDLc, and triglycerides.

### Lipids and IQ

Information on lipids and IQ was available for 3713 children (Table 2). The IQ decreased by .93 points for each mmol/L increase in LDLc (*p* = .04), but this attenuated to .58 points (*p* = .15) after adjustment for maternal education and household social class. The 2254 children who had *APOE* information demonstrated a similar pattern. Associations of quartiles of lipid measures with IQ and lipids (as continuous measures and quartiles) with other cognitive function measures are shown in Tables S3–S5 in Supplement 1.

Individuals in the second and fourth quartiles of LDLc demonstrated lower IQ scores than those in the lowest quartile, but these associations did attenuate after adjustment for maternal education and social class (Table S3 in Supplement 1). There was some evidence that being in the highest quartiles of either total cholesterol or LDLc was associated with lower scores in key stage 2 math and the nonword repetition memory test (Table S5 in Supplement 1). These associations remained after adjustment for maternal education and household socioeconomic position.

### Cognitive Function

There was little evidence to suggest that IQ, WORD, and WOLD test results were associated with *APOE* genotype (Table 3). However, on each measure, children who carried ε2/2 and ε4/4 genotypes tended to have slightly higher scores. For example, total IQ was 3.6 points higher for children who carried ε2/2 compared with those who carried ε3/3 and 2.6 points higher for ε4/4 compared with ε3/3. Memory scores, including nonword repetition task results, were not associated with *APOE* genotype (Table 4). The SATS scores were essentially unrelated to *APOE* genotype (Table 5 and Table S6 in Supplement 1).

However, there was a consistent pattern that ε2/2 and ε4/4 girls had higher IQ scores (from 3 to 7 points) compared with ε3/3 girls (Table S7 in Supplement 1). These genotypes were also associated with SATS scores among girls (Table S8 in Supplement 1); this pattern was less evident among boys.

## Discussion

We have undertaken a composite analysis including *APOE* genotype and serum lipid, IQ, and educational measures in a large population-based sample of children. Lipid profiles differed by *APOE* genotype in characteristic patterns corresponding with the wider literature, which has largely focused on adults (21). There was strong statistical evidence for these genotype-specific differences in total cholesterol, HDLc, triglycerides, and calculated LDLc, and the data define with high precision the population patterns for this age group and era in the UK. By contrast, despite known associations of *APOE* genotype with cognitive decline in adults and Alzheimer's disease, no differences were evident for the main genotype groups for IQ, memory, and educational measures of children, although there were possible differences for rare genotype subgroups.

The total and LDLc values for the different genotypes in our cohort (age 9) are similar although slightly lower than the average values observed in a population based-sample of 3–18-year-olds in Finland in 1980 and a sample of 11-year-old Greek schoolchildren (41,42). A study on the same Finnish population found that the characteristic differences in lipid levels by *APOE* genotype were present at age 3 but not in newborns, suggesting that these associations develop in the first few years of life (43). For HDLc level, genotype differences were less marked in our sample than differences in LDLc and total cholesterol, but there was clear evidence of an increase with ε2 alleles and a decrease with ε4 alleles, which was not obvious in the Finnish population (41). The lipid data act as a positive control for our genotyping and database operations, confirming that the absence of association with cognitive measures in our study does not reflect technical limitations.

Because of the wide range of cognitive function measures investigated within ALSPAC (both clinic measures and the results of nationally administered tests), our study provides good evidence that having an ε4 allele is not detrimental to cognitive performance in childhood or adolescence. This study was sufficiently powered to detect differences of 1.9 and 2.0 IQ points in ε4/ε3 and ε2/ε3 genotypes, respectively, compared with ε3 homozygotes but only differences of 5.1 and 8.5 IQ points in ε4 and ε2 homozygotes, respectively. Furthermore, it might be expected that the ε4 allele would have an additive effect on cognitive function (i.e., having one ε4 allele has a weaker effect than having two), but the effects are in the same direction—as is demonstrated for both lipids and LOAD (21,44)—but we did not observe this pattern in our results. In addition, the ε2 allele did not exert any effect in the common ε2/3 heterozygote group. This lack of association with cognitive function measures in ALSPAC is consistent with the findings of previous smaller studies in children of a similar age (24,25) and suggests that the preclinical effects of Alzheimer's disease do not start in childhood. The human ancestral allele for *APOE* is widely accepted to be ε4, the sequence observed in other primates and from which two sequential mutations at CpG sites representing arginine codons 112 and 158 have respectively generated alleles ε3 and ε2, under putative positive selection (45). The maximal biological contrast should therefore be between ε4 and ε2, and the absence of difference in our study argues against cognitive function in earlier life being a selective pressure.

It might be, as is suggested by the results of studies of normal cognitive aging in older adults, that *APOE* is only associated with certain domains of cognitive function, and so general measures such as IQ and attainment tests have not captured specific differences. However, memory function, one of the domains that has most commonly been found to be associated with *APOE* genotype in older people (12,46), did not differ between *APOE* genotype groups at ages 8 and 10.

The minor homozygote groups (ε4/4 and ε2/2) displayed the greatest magnitude associations (both positive) with IQ, and ε2/2 homozygotes also displayed the greatest magnitude associations with key stage 2 test scores, especially in girls (Tables S7 and S8 in Supplement 1). Given the small numbers in these groups, the confidence intervals are wide and are consistent with there being no effect. However, the results might suggest some cognitive advantage in these groups. Such a scenario could be biologically plausible if lipid levels during childhood affect brain development, a hypothesis that has been put forward with regard to the ε4 allele and cholesterol levels and for which there is some evidence in the literature (28,29). Lipid levels are affected both by environmental factors, such as diet, and by genetic variants such as *APOE*. The *APOE* genotype therefore acts as a genocopy for environment-affected lipid levels. Among rare lipoprotein disorders, microsomal triglyceride transfer protein genocopies the effects on the central nervous system of both genetic- and environment-driven forms of vitamin E deficiency (47–49). Higher triglyceride levels are observed in both ε4 and ε2 carriers compared with ε3/3 (Table 1) and numerous important substances such as polyunsaturated fatty acids (50), palmitate (51), fish oils (i.e., N-3 fatty acids) (52), and fat-soluble vitamins such as vitamin E (47)—which have recognized importance in brain development—are also carried by lipoprotein particles. Further studies would be required with larger numbers in the minor homozygote groups to obtain robust conclusions for them.

Our genetic observations are consistent with the attenuation of the association between LDLc and IQ (Table 2), after adjustment for the confounding factors maternal education and socioeconomic class. Previous studies in young and middle-age adults (53,54) have suggested associations between lipid measures and intellectual performance. However, no consistency between phenotypes (which included immediate and delayed word recall, fluid and crystallized intelligence) or studies has emerged. We have analyzed by quartiles of lipid measures (Tables S3 and S5 in Supplement 1) to look for U-shaped relationships as well as by regression on the assumption of a monotonic quantitative association (Table 2 and Table S4 in Supplement 1). Overall, the most apparent positive findings (e.g., lower non–word repetition score in higher LDLc quartiles) were attenuated after adjustment for confounding factors and residual effects were inconsistent (e.g., lower IQ in second and fourth quartiles of LDLc compared with the first and third). It would be possible in the future, with a large number of genetic variants influencing LDLc (55), to use Mendelian randomization tests (56) of whether there is causal association between circulating LDLc and cognitive function.

We found some evidence of an interaction between genotype and gender in the IQ analyses, which raises the possibility that *APOE* genotype might have a different effect on cognitive function in male subjects and female subjects. Mortensen and Hogh (9) found a decline in IQ scores in ε4 carriers from age 70 to 80 in women but not men, although the study sample comprised only 163 people. However, although associations between *APOE* genotype were stronger when our analyses were stratified by gender than in the sample as a whole, there was no consistent pattern across the different cognitive measures, and evidence for these associations would not remain after Bonferroni correction for the number of statistical tests performed. Thus, support for a gender difference in the association between cognitive measures and *APOE* is limited.

In conclusion, although the estimates for the homozygote groups are less precise, due to relatively small numbers we can be confident that—at least for the three major genotype groups (ε3/ε3, ε3/ε4, and ε2/ε3) that represent 94% of this population—*APOE* genotype has no major influence on cognition in childhood and adolescence. However, given the strong associations with lipid profiles in these children, *APOE* genotype should be considered important in the context of the origins of cardiovascular disease.

## Figures and Tables

**Table 1:** Lipids by *APOE* Genotype

Genotype	Total Cholesterol (mmol/L)					LDLc (mmol/L)				HDLc (mmol/L)				Triglyceride^a^ (mmol/L)				
	*n*	Mean (SD)	Coeff^b^	95% CI	*p*^c^	Mean (SD)	Coeff^b^	95% CI	*p*^c^	Mean (SD)	Coeff^b^	95% CI	*p*^c^	Geometric Mean	(IQR)	Coeff^b^	95% CI	*p*^c^
ε2/ε2	26	3.61 (.65)	−.64	(−.89, −.40)		1.37 (.66)	−.96	(−1.17, −.75)		1.55 (.44)	.15	(.03, .27)		1.35	(.95, 1.86)	1.34	(1.14, 1.58)	
ε2/ε3	382	3.95 (.58)	−.29	(−.36, −.22)		1.95 (.48)	−.38	(−.44, −.32)		1.48 (.33)	.08	(.04, .11)		1.04	(.79, 1.34)	1.04	(.99, 1.09)	
ε4/ε2	74	4.17 (.65)	−.07	(−.22, .07)		2.14 (.54)	−.2	(−.33, −.07)		1.45 (.36)	.05	(−.02, .12)		1.14	(.86, 1.53)	1.14	(1.03, 1.26)	
ε3/ε3	1626	4.25 (.65)	0	—		2.34 (.57)	0	—		1.40 (.30)	0	—		1	(.75, 1.33)	1	—	
ε3/ε4	709	4.39 (.62)	.14	(.09, .20)		2.51 (.56)	.16	(.12, .21)		1.35 (.29)	−.05	(−.08, −.03)		1.07	(.78, 1.42)	1.07	(1.04, 1.11)	
ε4/ε4	58	4.65 (.57)	.4	(.23, .56)	<.0001	2.72 (.53)	.37	(.23, .52)	<.0001	1.31 (.22)	−.09	(−.17, −.01)	<.0001	1.26	(1.00, 1.60)	1.25	(1.12, 1.40)	<.0001

APOE, apolipoprotein E; LDLc, low-density lipoprotein cholesterol; HDLc, high-density lipoprotein cholesterol; CI, confidence interval; IQR, interquartile range.

a Coefficient represents the ratio of geometric means, because triglyceride data are log-transformed.

b Coefficient from linear regression adjusted for age and gender with ε3/ε3 genotype as reference group.

c The *p* value for heterogeneity from analysis of covariance model.

**Table 2:** Associations of Plasma Lipids with Total IQ

	Coeff^a^ (IQ points)	Age, Gender-Adjusted		Coeff^a^ (IQ points)	All Adjusted^b^	
		95% CI	*p*		95% CI	*p*
All Children, *n* = 3713						
Total cholesterol (mmol/L)	−.69	(−1.46, .09)	.08	−.45	(−1.17, .26)	.22
LDLc (mmol/L)	−.92	(−1.79, −.06)	.04	−.59	(−1.38, .21)	.15
HDLc (mmol/L)	−.07	(−1.74, 1.60)	.93	−.27	(−1.82, 1.27)	.73
Triglyceride^c^ (mmol/L)	.54	(−.64, 1.73)	.37	.35	(−.74, 1.44)	.53
Children with *APOE* Genotype Data, *n* = 2254						
Total cholesterol (mmol/L)	−.78	(−1.78, .23)	.13	−.70	(−1.64, .23)	.14
LDLc (mmol/L)	−.98	(−2.10, .15)	.09	−.85	(−1.89, .20)	.11
HDLc (mmol/L)	−.79	(−2.95, 1.38)	.48	−.64	(−2.65, 1.37)	.53
Triglyceride^c^ (mmol/L)	1.15	(−.37, 2.67)	.14	.62	(−.80, 2.03)	.39

Abbreviations as in Table 1.

a Coefficients from linear regression represent increase in IQ points/mmol/L increase in lipid measure.

b Age, gender, maternal education, household social class adjusted.

c Triglyceride is log-transformed.

**Table 3:** Associations of *APOE* Genotype with IQ, WORD, and WOLD Tests

	Coeff^a^	95% CI	*p*^b^
Total IQ^c^, *n* = 3925			
ε2/ε2	3.62	(−2.20, 9.44)	
ε2/ε3	.29	(−1.24, 1.83)	
ε2/ε4	.85	(−2.34, 4.04)	
ε3/ε3	—	—	
ε3/ε4	.13	(−1.10, 1.35)	
ε4/ε4	2.61	(−.87, 6.09)	.58
Verbal IQ^c^, *n* = 3938			
ε2/ε2	3.86	(−2.04, 9.77)	
ε2/ε3	−.07	(−1.63, 1.48)	
ε2/ε4	.30	(−2.94, 3.53)	
ε3/ε3	—	—	
ε3/ε4	−.37	(−1.61, .88)	
ε4/ε4	2.24	(−1.29, 5.77)	.58
Performance IQ^c^, *n* = 3940			
ε2/ε2	2.78	(−3.29, 8.85)	
ε2/ε3	.83	(−.76, 2.43)	
ε2/ε4	1.27	(−2.06, 4.60)	
ε3/ε3	—	—	
ε3/ε4	.93	(−.35, 2.21)	
ε4/ε4	2.41	(−1.22, 6.04)	.46
WORD^d^, *n* = 4391			
ε2/ε2	1.46	(−1.59, 4.50)	
ε2/ε3	−.31	(−1.15, .52)	
ε2/ε4	−.44	(−2.21, 1.33)	
ε3/ε3	—	—	
ε3/ε4	−.27	(−.94, .40)	
ε4/ε4	1.02	(−.92, 2.96)	.64
WOLD^d^, *n* = 3940			
ε2/ε2	.36	(−.35, 1.06)	
ε2/ε3	−.03	(−.21, .15)	
ε2/ε4	.08	(−.29, .46)	
ε3/ε3	—	—	
ε3/ε4	−.06	(−.21, .09)	
ε4/ε4	.28	(−.14, .69)	.56

APOE, apolipoprotein E; WORD, Wechsler Objective Reading Dimensions; WOLD, Wechsler Objective Language Dimensions; CI, confidence interval.

a From linear regression adjusted for gender and the age of child in months.

b The *p* values for heterogeneity from analysis of covariance model.

c Coefficients represent change in IQ score in IQ points.

d Coefficients represent point changes in WORD and WOLD scores.

**Table 4:** Scores in Memory Tests at Ages 8 and 10 by *APOE* Genotype

	Coefficient^a^	95% CI	*p*^b^
NonWord Repetition Task, *n* = 3937			
ε2/ε2	.43	(.49, 1.35)	
ε2/ε3	−.06	(−.30, .18)	
ε2/ε4	−.08	(−.58, .41)	
ε3/ε3	—	—	
ε3/ε4	−.09	(−.28, .11)	
ε4/ε4	−.08	(−.62, .46)	.86
Counting Span Task Global Score, *n* = 3667			
ε2/ε2	−.83	(−3.56, 1.90)	
ε2/ε3	.02	(−.73, .77)	
ε2/ε4	.30	(−1.29, 1.89)	
ε3/ε3	—	—	
ε3/ε4	.32	(−.28, .92)	
ε4/ε4	.98	(−.79, 2.75)	.75
Counting Span Task Span Score, *n* = 3667			
ε2/ε2	−.11	(−.42, .20)	
ε2/ε3	−.001	(−.08, .08)	
ε2/ε4	.02	(−.16, .20)	
ε3/ε3	—	—	
ε3/ε4	.05	(−.02, .12)	
ε4/ε4	.18	(−.02, .37)	.35

Nonword repetition task from age 8 clinic, counting span task from age 10 clinic. APOE, apolipoprotein E; CI, confidence interval.

a Adjusted for age and gender.

b The *p* values for heterogeneity from analysis of covariance model.

**Table 5:** Key Stage 2 and 3 Test Scores by *APOE* Genotype

	Key Stage 2					Key Stage 3				
		*n*	Coeff^a^	95% CI	*p*^b^		*n*	Coeff^a^	95% CI	*p*^b^
English	*n* = 5245					*n* = 4487				
	ε2/ε2	46	3.26	(−1.22, 7.74)		ε2/ε2	42	3.33	(−1.85, 8.50)	
	ε2/ε3	704	−.76	(−2.02, .51)		ε2/ε3	618	.51	(−.98, 2.00)	
	ε2/ε4	141	−.39	(−2.98, 2.21)		ε2/ε4	122	.74	(−2.35, 3.82)	
	ε3/ε3	3019	—	—		ε3/ε3	2566	—	—	
	ε3/ε4	1218	−.64	(−1.66, .38)		ε3/ε4	1036	.06	(−1.16, 1.29)	
	ε4/ε4	117	−.38	(−3.22, 2.46)	.45	ε4/ε4	103	.32	(−3.02, 3.67)	.83
Math	*n* = 5263					*n* = 4607				
	ε2/ε2	45	3.79	(−2.29, 9.87)		ε2/ε2	43	1.27	(−5.20, 7.73)	
	ε2/ε3	709	−.59	(−2.28, 1.10)		ε2/ε3	632	−1.38	(−3.25, .48)	
	ε2/ε4	140	−1.06	(−4.56, 2.44)		ε2/ε4	125	−.99	(−4.84, 2.87)	
	ε3/ε3	3031	—	—		ε3/ε3	2644	—	—	
	ε3/ε4	1222	−.78	(−2.15, .59)		ε3/ε4	1054	.22	(−1.31, 1.75)	
	ε4/ε4	116	−2.02	(−5.85, 1.81)	.52	ε4/ε4	109	−.13	(−4.24, 3.98)	.72
Science	*n* = 5310					*n* = 4635				
	ε2/ε2	44	3.32	(−.22, 6.86)		ε2/ε2	43	−2.86	(−10.05, 4.34)	
	ε2/ε3	713	−.47	(−1.43, .50)		ε2/ε3	633	.98	(−1.10, 3.05)	
	ε2/ε4	141	.04	(−1.97, 2.05)		ε2/ε4	127	.89	(−3.37, 5.14)	
	ε3/ε3	3067	—	—		ε3/ε3	2661	—	—	
	ε3/ε4	1228	−.52	(−1.30, .27)		ε3/ε4	1063	1.31	(−.39, 3.01)	
	ε4/ε4	117	.46	(−1.73, 2.65)	.28	ε4/ε4	108	2.89	(−1.70, 7.49)	.46

APOE, apolipoprotein E; CI, confidence interval.

a From linear regression adjusted for gender and age at testing in months.

b The *p* values for heterogeneity from analysis of covariance model.
