# Supplementary material for: IQ, Educational Attainment, Memory and Plasma Lipids: Associations with Apolipoprotein E Genotype in 5995 Children
Source: Biol Psychiatry. 2011 Jul 15;70(2):152–8. doi: 10.1016/j.biopsych.2010.10.033 (PMC3130925; doi:10.1016/j.biopsych.2010.10.033)
Supplement: Supplement 1 [file mmc1.pdf]

Supplemental Information

Table S1. Lipid levels by *APOE* genotype in males

		Total cholesterol (mmol/l)				LDL cholesterol (mmol/l)				HDL cholesterol (mmol/l)				Triglyceride[c] (mmol/l)				
**Genotype**	**N**	**Mean (SD)**	**Coeff[a]**	**95% CI**	**p[b]**	**Mean (SD)**	**Coeff[a]**	**95% CI**	**p[b]**	**Mean (SD)**	**Coeff[a]**	**95% CI**	**p[b]**	**Geometric mean**	**(IQR)**	**Coeff[a]**	**95% CI**	**p[b]**
ε2/ε2	14	3.52 (0.64)	-0.65	(-0.99 to -0.32)		1.33 (0.68)	-0.92	(-1.21 to -0.63)		1.56 (0.51)	0.13	(-0.03 to 0.30)		1.30	(0.94-1.86)	1.34	(1.06 to 1.68)	
ε2/ε3	231	3.92 (0.55)	-0.26	(-0.35 to -0.17)		1.89 (0.46)	-0.36	(-0.44 to -0.28)		1.52 (0.31)	0.08	(0.04 to 0.13)		1.03	(0.77-1.34)	1.06	(0.99 to 1.13)	
ε2/ε4	41	4.06 (0.75)	-0.12	(-0.32 to 0.08)		2.02 (0.61)	-0.23	(-0.40 to -0.06)		1.48 (0.39)	0.05	(-0.05 to 0.15)		1.07	(0.91-1.27)	1.11	(0.97 to 1.27)	
ε3/ε3	885	4.18 (0.65)	0	-		2.26 (0.56)	0	-		1.43 (0.31)	0	-		0.97	(0.72- 1.30)	0	-	
ε3/ε4	380	4.36 (0.64)	0.19	(0.11 to 0.26)		2.46 (0.56)	0.20	(0.14 to 0.27)		1.39 (0.31)	-0.04	(-0.08 to -0.003)		1.02	(0.75-1.35)	1.05	(1.00 to 1.11)	
ε4/ε4	30	4.62 (0.57)	0.44	(0.21 to 0.67)	<0.0001	2.73 (0.49)	0.47	(0.27 to 0.67)	<0.0001	1.27 (0.23)	-0.16	(-0.27 to -0.04)	<0.0001	1.26	(0.95-1.77)	1.30	(1.11 to 1.53)	0.001

Table S2. Lipid levels by *APOE* genotype in females

		Total cholesterol (mmol/l)				LDL cholesterol (mmol/l)				HDL cholesterol (mmol/l)				Triglyceride[c] (mmol/l)				
**Genotype**	**N**	**Mean (SD)**	**Coeff[a]**	**95% CI**	**p[b]**	**Mean (SD)**	**Coeff[a]**	**95% CI**	**p[b]**	**Mean (SD)**	**Coeff[a]**	**95% CI**	**p[b]**	**Geometric mean**	**(IQR)**	**Coeff[a]**	**95% CI**	**p[b]**
ε2/ε2	12	3.70 (0.67)	-0.63	(-0.97 to -0.28)		1.44 (0.66)	-1.00	(-1.32 to -0.69)		1.54 (0.36)	0.18	(0.008 to 0.34)		1.40	(0.99-1.89)	1.35	(1.06 to 1.71)	
ε2/ε3	151	4.00 (0.62)	-0.34	(-0.44 to -0.23)		2.04 (0.51)	-0.40	(-0.50 to -0.31)		1.43 (0.35)	0.06	(0.01 to 0.12)		1.06	(0.81-1.4)	1.02	(0.95 to 1.10)	
ε2/ε4	33	4.31 (0.46)	-0.02	(-0.23 to 0.20)		2.30 (0.39)	-0.15	(-0.34 to 0.04)		1.41 (0.33)	0.05	(-0.06 to 0.15)		1.22	(1.05-1.41)	1.17	(1.01 to 1.36)	
ε3/ε3	741	4.33 (0.64)	0	-		2.45 (0.56)	0	-		1.36 (0.30)	0	-		1.04	(0.77-1.35)	0	-	
ε3/ε4	329	4.42 (0.60)	0.09	(0.005 to 0.17)		2.56 (0.55)	0.12	(0.05 to 0.19)		1.29 (0.27)	-0.07	(-0.11 to -0.03)		1.13	(0.84-1.51)	1.09	(1.03 to 1.15)	
ε4/ε4	28	4.67 (0.58)	0.34	(0.10 to 0.57)	<0.0001	2.71 (0.57)	0.26	(0.05 to 0.47)	<0.0001	1.35 (0.21)	-0.02	(-0.13 to 0.10)	<0.0001	1.25	(1.01-1.50)	1.20	(1.03 to 1.41)	0.0006

[a] Coefficient from linear regression adjusted for age with ε3/ε3 genotype as reference group

[b] P values for heterogeneity from analysis of covariance model

[c] Triglycerides are log transformed so coefficients represent ratio of geometric means

Table S3. Associations of lipids (in quartiles) with IQ

All children (N = 3713)

	Age, sex adjusted				All adjusted[b]			
	Quartile	Coeff[a] (IQ points)	95% CI	P	Quartile	Coeff[a] (IQ points)	95% CI	P
Total cholesterol	1	-	-	-	1	-	-	-
	2	-0.48	(-1.93, 0.97)	0.52	2	-0.13	(-1.47, 1.21)	0.85
	3	-0.31	(-1.75, 1.13)	0.67	3	0.26	(-1.07, 1.59)	0.70
	4	-1.03	(-2.49, 0.42)	0.16	4	-0.60	(-1.94, 0.74)	0.38
LDL	1	-	-	-	1	-	-	-
	2	-1.83	(-3.29, -0.38)	0.01	2	-1.17	(-2.51, 0.17)	0.09
	3	-0.64	(-2.10, 0.82)	0.39	3	-0.21	(-1.56, 1.14)	0.76
	4	-1.52	(-2.99, -0.05)	0.04	4	-0.83	(-2.18, 0.53)	0.23
HDL	1	-	-	-	1	-	-	-
	2	0.61	(-0.82, 2.05)	0.40	2	0.14	(-1.18, 1.46)	0.84
	3	-0.0005	(-1.46, 1.46)	1.00	3	-0.23	(-1.57, 1.12)	0.74
	4	0.31	(-1.15, 1.77)	0.68	4	-0.05	(-1.40, 1.30)	0.94
Triglycerides	1	-	-	-	1	-	-	-
	2	1.45	(-0.004, 2.90)	0.05	2	0.84	(-0.50, 2.18)	0.22
	3	0.75	(-0.70, 2.20)	0.31	3	0.20	(-1.14, 1.54)	0.77
	4	0.62	(-0.84, 2.08)	0.40	4	0.44	(-0.91, 1.79)	0.52

Table S3 continued

Children with *APOE* genotype available (N = 2254)

	Age, sex adjusted				All adjusted[b]			
	Quartile	Coeff (IQ points)[a]	95% CI	P	Quartile	Coeff (IQ points)[a]	95% CI	P
Total cholesterol	1	-	-	-	1	-	-	-
	2	-1.58	(-3.42, 0.25)	0.09	2	-1.11	(-2.82, 0.59)	0.20
	3	-0.80	(-2.64, 1.04)	0.39	3	-0.38	(-2.09, 1.32)	0.66
	4	-1.79	(-3.66, 0.07)	0.06	4	-1.55	(-3.28, 0.18)	0.08
LDL	1	-	-	-	1	-	-	-
	2	-3.54	(-5.38, -1.71)	<0.001	2	-2.86	(-4.57, -1.16)	0.001
	3	-1.51	(-3.38, 0.36)	0.11	3	-1.26	(-2.99, 0.48)	0.16
	4	-2.51	(-4.38, -0.65)	0.01	4	-2.23	(-3.96, -0.50)	0.01
HDL	1	-	-	-	1	-	-	-
	2	0.32	(-1.53, 2.17)	0.74	2	-0.06	(-1.77, 1.66)	0.95
	3	-0.51	(-2.37, 1.35)	0.59	3	-0.36	(-2.09, 1.36)	0.68
	4	-0.60	(-2.47, 1.27)	0.53	4	-0.55	(-2.29, 1.19)	0.53
Triglycerides	1	-	-	-	1	-	-	-
	2	1.96	(0.09, 3.84)	0.04	2	0.83	(-0.92, 2.58)	0.35
	3	1.60	(-0.24, 3.45)	0.09	3	0.44	(-1.28, 2.16)	0.61
	4	1.34	(-0.54, 3.22)	0.16	4	0.56	(-1.19, 2.31)	0.53

[a] Coefficients represent change in IQ points for each quartile of lipid compared to the lowest quartile

[b]Age, sex, maternal education, household social class adjusted

Quartiles (all in mmol/l): Total cholesterol: <3.83, 3.84-4.23, 4.24-4.67, >4.67; LDL cholesterol: <1.96, 1.96-2.31, 2.31-2.71, >2.71; HDL cholesterol: <1.19, 1.2-1.38, 1.39-1.60, 1.61-2.88 Triglycerides: <0.77, 0.77-1.01, 1.02-1.38, >1.38

Table S4. Associations of lipids with cognitive function measures

	Age, sex adjusted				Fully adjusted[c]			
	**N**	**Coeff[b]**	**95% CI**	**p**	**N**	**Coeff[b]**	**95% CI**	**p**
**Key stage 3 English**								
Total cholesterol (mmol/l)	3270	-0.76	(1.60, 0.08)	0.07	3270	-0.49	(-1.27, 0.28)	0.21
LDL cholesterol (mmol/l)	3270	-1.05	(-1.97, -0.12)	0.03	3270	-0.72	(-1.58, 0.13)	0.10
HDL cholesterol (mmol/l)	3270	-0.69	(-2.49, 1.11)	0.45	3270	-0.81	(-2.48, 0.86)	0.34
Triglyceride[a] (mmol/l)	3270	1.25	(-0.03, 2.52)	0.06	3270	1.17	(-0.01, 2.35)	0.05
**Key stage 3 Maths**								
Total cholesterol (mmol/l)	3305	-1.08	(-2.17, 0.01)	0.05	3305	-0.80	(-1.84, 0.24)	0.13
LDL cholesterol (mmol/l)	3305	-1.16	(-2.36, 0.04)	0.06	3305	-0.80	(-1.95, 0.35)	0.17
HDL cholesterol (mmol/l)	3305	-0.34	(-2.69, 2.00)	0.77	3305	-0.53	(-2.77, 1.71)	0.64
Triglyceride[a] (mmol/l)	3305	0.31	(-1.35, 1.97)	0.71	3305	0.26	(-1.33, 1.85)	0.75
**Key stage 3 Science**								
Total cholesterol (mmol/l)	3322	-0.70	(-1.89, 0.48)	0.25	3322	-0.53	(-1.71, 0.64)	0.38
LDL cholesterol (mmol/l)	3322	-0.54	(-1.85, 0.78)	0.42	3322	-0.31	(-1.61, 0.98)	0.64
HDL cholesterol (mmol/l)	3322	-1.05	(-3.60, 1.49)	0.42	3322	-1.21	(-3.73, 1.32)	0.35
Triglyceride[a] (mmol/l)	3322	0.44	(-1.38, 2.25)	0.64	3322	0.43	(-1.37, 2.22)	0.64
**Key stage 2 English**								
Total cholesterol (mmol/l)	3772	-0.47	(-1.15, 0.21)	0.18	3772	-0.27	(-0.91, 0.36)	0.40
LDL cholesterol (mmol/l)	3772	-0.68	(-1.44, 0.07)	0.08	3772	-0.41	(-1.11, 0.30)	0.26
HDL cholesterol (mmol/l)	3772	0.55	(-0.92, 2.01)	0.46	3772	0.31	(-1.06, 1.68)	0.65
Triglyceride[a] (mmol/l)	3772	0.07	(-0.97, 1.11)	0.89	3772	-0.02	(-0.99, 0.95)	0.97

Table S4 continued

**Key stage 2 Maths**								
Total cholesterol (mmol/l)	3777	-1.21	(2.14, 0.29)	0.01	3777	-0.93	(-1.80, -0.06)	0.04
LDL cholesterol (mmol/l)	3777	-1.25	(-2.27, -0.22)	0.02	3777	-0.89	(-1.86, 0.07)	0.07
HDL cholesterol (mmol/l)	3777	-0.28	(-2.27, 1.71)	0.78	3777	-0.46	(-2.33, 1.41)	0.63
Triglyceride[a] (mmol/l)	3777	-0.28	(-1.70, 1.14)	0.70	3777	-0.38	(-1.71, 0.96)	0.58
**Key stage 2 Science**								
Total cholesterol (mmol/l)	3790	-0.57	(-1.08, -0.07)	0.03	3790	-0.42	(-0.89, 0.06)	0.08
LDL cholesterol (mmol/l)	3790	-0.57	(-1.13, 0.00)	0.05	3790	-0.36	(-0.88, 0.17)	0.18
HDL cholesterol (mmol/l)	3790	-0.60	(-1.69, 0.49)	0.28	3790	-0.68	(-1.70, 0.34)	0.19
Triglyceride[a] (mmol/l)	3790	0.05	(-0.55, 0.65)	0.87	3790	0.28	(-0.44, 1.01)	0.44
**Working memory global score**								
Total cholesterol (mmol/l)	3582	0.004	(-0.37, 0.38)	0.98	3582	0.07	(-0.30, 0.44)	0.70
LDL cholesterol (mmol/l)	3582	-0.11	(-0.53, 0.31)	0.60	3582	-0.01	(-0.43, 0.40)	0.94
HDL cholesterol (mmol/l)	3582	0.26	(-0.56, 1.08)	0.53	3582	0.21	(-0.59, 1.01)	0.61
Triglyceride[a] (mmol/l)	3582	0.08	(-0.50, 0.66)	0.78	3582	0.04	(-0.53, 0.61)	0.89
**Non word repetition**								
Total cholesterol (mmol/l)	3736	-0.17	(0.29, 0.05)	0.01	3736	-0.14	(-0.26, -0.02)	0.02
LDL cholesterol (mmol/l)	3736	-0.18	(0.32, 0.05)	0.01	3736	-0.14	(-0.27, -0.01)	0.03
HDL cholesterol (mmol/l)	3736	-0.07	(-0.33, 0.19)	0.58	3736	-0.10	(-0.35, 0.16)	0.46
Triglyceride[a] (mmol/l)	3736	-0.05	(-0.23, 0.13)	0.59	3736	-0.06	(-0.24, 0.12)	0.49

[a] Triglyceride is log transformed

[b]Coefficients from linear regression represent increase in IQ points per mmol/l increase in lipid measure

[c]Age, sex, maternal education, household social class adjusted

Table S5. Associations of lipids (in quartiles) with cognitive function measures

		Age, sex adjusted					All adjusted[b]				
		**Quartile**	**N**	**Coeff [a]**	**95% CI**	**P**	**Quartile**	**N**	**Coeff [a]**	**95% CI**	**P**
Key stage 3											
**English**	Total Cholesterol	1	812	-	-	-	1	812	-	-	-
		2	815	-0.98	(-2.55, 0.58)	0.22	2	815	-0.47	(-1.91, 0.98)	0.53
		3	819	-0.74	(-2.31, 0.82)	0.35	3	819	-0.19	(-1.64, 1.26)	0.8
		4	824	-1.15	(-2.71, 0.42)	0.15	4	824	-0.65	(-2.10, 0.80)	0.38
	LDL	1	781	-	-	-	1	781	-	-	-
		2	818	-1.02	(-2.60, 0.56)	0.21	2	818	-0.53	(-1.99, 0.93)	0.48
		3	852	-0.08	(-1.65, 1.49)	0.92	3	852	0.18	(-1.28, 1.64)	0.81
		4	819	-1.19	(-2.78, 0.40)	0.14	4	819	-0.63	(-2.11, 0.84)	0.4
		1	854	-	-	-	1	854	-	-	-
	HDL	2	826	0.38	(-1.16, 1.92)	0.63	2	826	0.22	(-1.21, 1.64)	0.77
		3	792	-0.95	(-2.51, 0.61)	0.23	3	792	-0.67	(-2.12, 0.77)	0.36
		4	798	-0.53	(-2.09, 1.03)	0.5	4	798	-0.79	(-2.24, 0.65)	0.28
		1	856	-	-	-	1	856	-	-	-
	Triglycerides	2	815	0.38	(-1.16, 1.93)	0.63	2	815	-0.13	(-1.56, 1.30)	0.85
		3	808	2.22	(0.67, 3.77)	0.01	3	808	1.62	(0.18, 3.05)	0.03
		4	791	0.72	(-0.83, 2.28)	0.36	4	791	0.60	(-0.84, 2.04)	0.42
**Maths**	Total Cholesterol	1	824	-	-	-	1	824	-	-	-
		2	819	0.52	(-1.51, 2.56)	0.61	2	819	1.06	(-0.89, 3.00)	0.29
		3	834	-0.71	(-2.74, 1.32)	0.49	3	834	-0.11	(-2.05, 1.84)	0.91
		4	828	-0.98	(-3.01, 1.06)	0.35	4	828	-0.48	(-2.42, 1.47)	0.63

	LDL	1	792	-	-	-	1	792	-	-	-
		2	827	-1.57	(-3.62, 0.48)	0.13	2	827	-1.01	(-2.97, 0.95)	0.31
		3	860	-0.15	(-2.19, 1.89)	0.88	3	860	0.18	(-1.78, 2.13)	0.86
		4	826	-2.00	(-4.07, 0.06)	0.06	4	826	-1.41	(-3.39, 0.56)	0.16
	HDL	1	869	-	-	-	1	869	-	-	-
		2	833	1.18	(-0.81, 3.18)	0.25	2	833	0.97	(-0.94, 2.88)	0.32
		3	802	0.59	(-1.43, 2.62)	0.57	3	802	0.87	(-1.07, 2.80)	0.38
		4	801	0.31	(-1.72, 2.34)	0.76	4	801	-0.04	(-1.98, 1.90)	0.97
	Triglycerides	1	857	-	-	-	1	857	-	-	-
		2	823	0.98	(-1.04, 2.99)	0.34	2	823	0.58	(-1.35, 2.51)	0.56
		3	825	1.10	(-0.92, 3.12)	0.28	3	825	0.69	(-1.24, 2.62)	0.48
		4	800	-0.04	(-2.07, 1.99)	0.97	4	800	-0.09	(-2.03, 1.85)	0.93
**Science**	Cholesterol	1	827	-	-	-	1	827	-	-	-
		2	823	-0.14	(-2.36, 2.08)	0.90	2	823	0.09	(-2.11, 2.29)	0.93
		3	839	-0.03	(-2.24, 2.19)	0.98	3	839	0.33	(-1.87, 2.52)	0.77
		4	833	-1.36	(-3.58, 0.86)	0.23	4	833	-1.09	(-3.29, 1.10)	0.33
	LDL	1	792	-	-	-	1	792	-	-	-
		2	835	-1.35	(-3.59, 0.89)	0.24	2	835	-0.99	(-3.21, 1.22)	0.38
		3	865	-1.28	(-3.51, 0.95)	0.26	3	865	-1.07	(-3.29, 1.14)	0.34
		4	830	-1.65	(-3.91, 0.62)	0.15	4	830	-1.29	(-3.52, 0.95)	0.26
	HDL	1	874	-	-	-	1	874	-	-	-
		2	832	0.31	(-1.87, 2.50)	0.78	2	832	0.23	(-1.93, 2.39)	0.84
		3	808	0.29	(-1.92, 2.50)	0.80	3	808	0.46	(-1.73, 2.64)	0.68
		4	808	-1.02	(-3.23, 1.19)	0.36	4	808	-1.26	(-3.45, 0.93)	0.26

	Triglycerides	1	864	-	-	-	1	864	-	-	-
		2	827	-0.41	(-2.61, 1.78)	0.71	2	827	-0.64	(-2.82, 1.53)	0.56
		3	830	2.05	(-0.15, 4.25)	0.07	3	830	1.77	(-0.40, 3.95)	0.11
		4	801	0.18	(-2.03, 2.39)	0.87	4	801	0.18	(-2.01, 2.37)	0.87
Key stage 2											
English	Cholesterol	1	966	-	-	-	1	966	-	-	-
		2	925	-0.91	(-2.18, 0.36)	0.16	2	925	-0.35	(-1.54, 0.83)	0.56
		3	945	-0.40	(-1.66, 0.87)	0.54	3	945	0.18	(-1.00, 1.36)	0.76
		4	936	-1.06	(-2.33, 0.21)	0.10	4	936	-0.69	(-1.88, 0.49)	0.25
	LDL	1	918	-	-	-	1	918	-	-	-
		2	949	-0.99	(-2.26, 0.29)	0.13	2	949	-0.57	(-1.77, 0.62)	0.35
		3	969	-0.39	(-1.67, 0.88)	0.55	3	969	-0.08	(-1.27, 1.12)	0.9
		4	936	-0.93	(-2.22, 0.36)	0.16	4	936	-0.53	(-1.73, 0.68)	0.39
	HDL	1	980	-	-	-	1	980	-	-	-
		2	959	1.27	(0.02, 2.52)	0.05	2	959	0.94	(-0.23, 2.11)	0.12
		3	919	-0.04	(-1.31, 1.23)	0.95	3	919	-0.01	(-1.20, 1.18)	0.99
		4	914	0.54	(-0.74, 1.81)	0.41	4	914	0.16	(-1.03, 1.35)	0.79
	Triglycerides	1	980	-	-	-	1	980	-	-	-
		2	944	0.54	(-0.72, 1.80)	0.40	2	944	-0.06	(-1.24, 1.12)	0.92
		3	942	0.99	(-0.27, 2.26)	0.12	3	942	0.39	(-0.79, 1.57)	0.52
		4	906	-0.01	(-1.28, 1.27)	0.99	4	906	-0.14	(-1.32, 1.05)	0.82
Maths	Cholesterol	1	961	-	-	-	1	961	-	-	-
		2	929	-0.76	(-2.49, 0.97)	0.39	2	929	-0.07	(-1.70, 1.55)	0.93
		3	944	-0.71	(-2.43, 1.02)	0.42	3	944	0.02	(-1.60, 1.64)	0.98
		4	943	-2.37	(-4.10, -0.65)	0.01	4	943	-1.84	(-3.46, -0.21)	0.03

	LDL	1	918	-	-	-	1	918	-	-	-
		2	947	-1.60	(-3.34, 0.14)	0.07	2	947	-1.05	(-2.69, 0.58)	0.21
		3	969	-1.45	(-3.19, 0.29)	0.10	3	969	-0.99	(-2.63, 0.65)	0.24
		4	943	-2.24	(-3.99, -0.48)	0.01	4	943	-1.72	(-3.37, -0.07)	0.04
	HDL	1	980	-	-	-	1	980	-	-	-
		2	955	0.79	(-0.91, 2.50)	0.36	2	955	0.42	(-1.18, 2.03)	0.61
		3	920	-0.25	(-1.98, 1.49)	0.78	3	920	-0.09	(-1.72, 1.53)	0.91
		4	922	0.42	(-1.32, 2.15)	0.64	4	922	0.04	(-1.59, 1.67)	0.96
	Triglycerides	1	978	-	-	-	1	978	-	-	-
		2	948	1.16	(-0.55, 2.88)	0.18	2	948	0.47	(-1.14, 2.09)	0.57
		3	944	0.75	(-0.98, 2.47)	0.40	3	944	-0.02	(-1.64, 1.60)	0.98
		4	907	-0.28	(-2.02, 1.45)	0.75	4	907	-0.38	(-2.01, 1.25)	0.65
Science	Cholesterol	1	967	-	-	-	1	967	-	-	-
		2	933	0.04	(-0.90, 0.99)	0.93	2	933	0.42	(-0.46, 1.30)	0.35
		3	948	-0.001	(-0.95, 0.94)	1.00	3	948	0.39	(-0.49, 1.27)	0.38
		4	942	-1.09	(-2.04, -0.14)	0.02	4	942	-0.79	(-1.67, 0.09)	0.08
	LDL	1	917	-	-	-	1	917	-	-	-
		2	959	-0.60	(-1.56, 0.35)	0.22	2	959	-0.28	(-1.17, 0.61)	0.54
		3	972	-0.26	(-1.21, 0.70)	0.60	3	972	0.04	(-0.85, 0.94)	0.92
		4	942	-1.06	(-2.03, -0.10)	0.03	4	942	-0.76	(-1.66, 0.14)	0.10
	HDL	1	986	-	-	-	1	986	-	-	-
		2	960	0.27	(-0.67, 1.20)	0.58	2	960	0.08	(-0.79, 0.95)	0.85
		3	920	-0.49	(-1.44, 0.46)	0.31	3	920	-0.43	(-1.32, 0.45)	0.34
		4	924	-0.04	(-0.99, 0.91)	0.93	4	924	-0.23	(-1.11, 0.66)	0.62

	Triglycerides	1	980	-	-	-	1	980	-	-	-
		2	954	1.20	(0.26, 2.14)	0.01	2	954	0.78	(-0.10, 1.65)	0.08
		3	946	0.81	(-0.13, 1.75)	0.09	3	946	0.35	(-0.53, 1.23)	0.43
		4	910	0.46	(-0.49, 1.41)	0.34	4	910	0.35	(-0.54, 1.23)	0.44
Working memory											
	Cholesterol	1	933	-	-	-	1	933	-	-	-
		2	879	0.01	(-0.70, 0.71)	0.99	2	879	0.16	(-0.53, 0.85)	0.65
		3	901	0.05	(-0.65, 0.75)	0.89	3	901	0.23	(-0.46, 0.92)	0.52
		4	869	-0.12	(-0.83, 0.59)	0.74	4	869	0.01	(-0.68, 0.71)	0.97
	LDL	1	890	-	-	-	1	890	-	-	-
		2	909	-0.07	(-0.78, 0.64)	0.85	2	909	0.07	(-0.62, 0.77)	0.83
		3	912	-0.29	(-1.00, 0.42)	0.42	3	912	-0.15	(-0.85, 0.55)	0.67
		4	871	-0.07	(-0.79, 0.65)	0.85	4	871	0.09	(-0.61, 0.80)	0.8
	HDL	1	928	-	-	-	1	928	-	-	-
		2	912	0.24	(-0.46, 0.94)	0.49	2	912	0.15	(-0.54, 0.83)	0.68
		3	891	0.28	(-0.43, 0.98)	0.44	3	891	0.24	(-0.45, 0.93)	0.5
		4	851	0.40	(-0.31, 1.12)	0.27	4	851	0.32	(-0.38, 1.02)	0.37
	Triglycerides	1	915	-	-	-	1	915	-	-	-
		2	911	-0.13	(-0.83, 0.57)	0.72	2	911	-0.29	(-0.98, 0.40)	0.41
		3	901	-0.19	(-0.89, 0.52)	0.6	3	901	-0.34	(-1.04, 0.35)	0.33
		4	855	0.03	(-0.69, 0.74)	0.94	4	855	-0.02	(-0.72, 0.68)	0.95
Non word repetition											
	Cholesterol	1	961	-	-	-	1	961	-	-	-
		2	907	-0.05	(-0.27, 0.18)	0.68	2	907	0	(-0.22, 0.21)	0.97
		3	947	-0.10	(-0.32, 0.12)	0.38	3	947	-0.04	(-0.26, 0.18)	0.71
		4	921	-0.29	(-0.52, -0.06)	0.01	4	921	-0.24	(-0.46, -0.02)	0.03

LDL	1	923	-	-	-	1	923	-	-	-
	2	939	-0.08	(-0.31, 0.14)	0.46	2	939	-0.02	(-0.24, 0.20)	0.83
	3	946	-0.13	(-0.35, 0.10)	0.28	3	946	-0.08	(-0.30, 0.14)	0.49
	4	928	-0.31	(-0.54, -0.08)	0.01	4	928	-0.24	(-0.46, -0.02)	0.04
HDL	1	947	-	-	-	1	947	-	-	-
	2	965	0.03	(-0.19, 0.25)	0.78	2	965	-0.01	(-0.23, 0.21)	0.92
	3	911	-0.02	(-0.24, 0.21)	0.9	3	911	-0.04	(-0.26, 0.18)	0.7
	4	913	0.001	(-0.23, 0.23)	0.99	4	913	-0.03	(-0.25, 0.19)	0.77
Triglycerides	1	942	-	-	-	1	942	-	-	-
	2	943	0.04	(-0.19, 0.26)	0.75	2	943	-0.01	(-0.23, 0.21)	0.91
	3	938	0.03	(-0.19, 0.26)	0.78	3	938	-0.02	(-0.24, 0.20)	0.88
	4	913	-0.12	(-0.34, 0.11)	0.31	4	913	-0.13	(-0.35, 0.09)	0.25

[a] Coefficients represent change in each cognitive function score for each quartile of lipid compared to the lowest quartile

[b]Age, sex, maternal education, household social class adjusted

Table S6. Attainment at key stage 1 by *APOE* genotype

	ε2/ε2		ε3/ε2		ε4/ε2		ε3/ε3		ε3/ε4		ε4/ε4		Total		
	**N**	**(%)**	**N**	**(%)**	**N**	**(%)**	**N**	**(%)**	**N**	**(%)**	**N**	**(%)**	**N**	**(%)**	**P[a]**
**Reading**															
Below average	6	(14.6)	93	(13.7)	12	(9.4)	391	(13.6)	156	(13.6)	16	(15.2)	674	(13.6)	
Average	23	(56.1)	357	(52.4)	70	(54.7)	1450	(50.5)	602	(52.5)	48	(45.7)	2550	(51.3)	
Above average	12	(29.27)	231	(33.9)	46	(35.9)	1029	(35.9)	389	(33.9)	41	(39.1)	1748	(35.2)	p = 0.82
**Writing**															
Below average	5	(12.2)	102	(15.0)	13	(10.2)	387	(13.5)	152	(13.2)	18	(17.1)	677	(13.6)	
Average	33	(80.5)	527	(77.5)	102	(79.7)	2236	(78.0)	902	(78.5)	74	(70.5)	3874	(78.0)	
Above average	3	(7.32)	51	(7.50)	13	(10.2)	244	(8.51)	95	(8.3)	13	(12.4)	419	(8.4)	p = 0.69
**Maths**															
Below average	3	(7.32)	70	(10.3)	5	(3.9)	255	(8.9)	97	(8.5)	11	(10.5)	441	(8.9)	
Average	34	(82.9)	433	(63.8)	84	(65.6)	1860	(64.9)	737	(64.2)	69	(65.7)	3217	(64.8)	
Above average	4	(9.76)	176	(25.9)	39	(30.5)	751	(26.2)	314	(27.4)	25	(23.8)	1309	(26.4)	p = 0.18

[a] p value from chi square test

Table S7. Associations of *APOE* genotype with IQ, stratified by sex

	Males				Females			
		Coeff[a]	95% CI	P[b]		Coeff[a]	95% CI	P[b]
**Total IQ**	**N = 2121**				**N = 1804**			
	ε2/ε2	2.67	(-5.31, 10.65)		ε2/ε2	5.02	(-3.45, 13.50)	
	ε2/ε3	0.49	(-1.59, 2.56)		ε2/ε3	0.16	(-2.12, 2.44)	
	ε2/ε4	2.70	(-1.70, 7.09)		ε2/ε4	-1.32	(-5.95, 3.31)	
	ε3/ε3	-	-		ε3/ε3	-	-	
	ε3/ε4	1.62	(-0.12, 3.35)		ε3/ε4	-1.63	(-3.35, 0.10)	
	ε4/ε4	-0.18	(-4.95, 4.58)	0.45	ε4/ε4	6.37	(1.29, 11.45)	0.03
**Verbal IQ**	**N = 2127**				**N = 1811**			
	ε2/ε2	2.52	(-5.71, 10.74)		ε2/ε2	5.75	(-2.68, 14.17)	
	ε2/ε3	-0.30	(-2.43, 1.84)		ε2/ε3	0.32	(-1.94, 2.57)	
	ε2/ε4	1.84	(-2.69, 6.36)		ε2/ε4	-1.53	(-6.13, 3.07)	
	ε3/ε3	-	-		ε3/ε3	-	-	
	ε3/ε4	0.55	(-1.23, 2.33)		ε3/ε4	-1.45	(-3.17, 0.26)	
	ε4/ε4	-1.14	(-6.05, 3.76)	0.88	ε4/ε4	6.73	(1.68, 11.78)	0.02
**Performance IQ**	**N = 2132**				**N = 1808**			
	ε2/ε2	2.95	(-5.33, 11.23)		ε2/ε2	2.74	(-6.17, 11.65)	
	ε2/ε3	1.67	(-0.48, 3.82)		ε2/ε3	-0.18	(-2.57, 2.21)	
	ε2/ε4	2.93	(-1.63, 7.49)		ε2/ε4	-0.66	(-5.53, 4.20)	
	ε3/ε3	-	-		ε3/ε3	-	-	
	ε3/ε4	2.72	(0.93, 4.52)		ε3/ε4	-1.16	(-2.97, 0.66)	
	ε4/ε4	0.97	(-3.97, 5.91)	0.06	ε4/ε4	4.43	(-0.91, 9.78)	0.39

[a] Coefficient from linear regression, adjusted for age at IQ testing with ε3/ε3 genotype as reference group

[b] P values for heterogeneity from analysis of covariance model

Table S8. Associations of *APOE* genotype with SATS scores, stratified by sex

	**Males**				**Females**		
	**Coeff[a]**	**95% CI**	**P[b]**		**Coeff[a]**	**95% CI**	**P[b]**
Key Stage 2							
**English**							
**N = 2739**				**N = 2506**			
ε2/ε2	3.96	(-2.15, 10.06)		ε2/ε2	2.40	(-4.20, 8.99)	
ε2/ε3	-0.34	(-2.08, 1.40)		ε2/ε3	-1.19	(-3.03, 0.65)	
ε2/ε4	1.16	(-2.38, 4.70)		ε2/ε4	-2.25	(-6.08, 1.58)	
ε3/ε3	-	-		ε3/ε3	-	-	
ε3/ε4	0.93	(-0.53, 2.39)		ε3/ε4	-2.31	(-3.73, -0.88)	
ε4/ε4	-2.19	(-6.25, 1.87)	0.39	ε4/ε4	1.54	(-2.41, 5.50)	0.02
**Maths**							
**N = 2762**				**N = 2501**			
ε2/ε2	4.34	(-3.78, 12.45)		ε2/ε2	3.03	(-6.17, 12.23)	
ε2/ε3	-0.98	(-3.28, 1.31)		ε2/ε3	-0.02	(-2.52, 2.49)	
ε2/ε4	-0.19	(-4.93, 4.54)		ε2/ε4	-2.10	(-7.30, 3.11)	
ε3/ε3	-	-		ε3/ε3	-	-	
ε3/ε4	0.47	(-1.47, 2.40)		ε3/ε4	-2.10	(-4.04, -0.16)	
ε4/ε4	-6.13	(-11.53, -0.73)	0.20	ε4/ε4	2.41	(-3.02, 7.83)	0.23
**Science**							
**N = 2796**				**N = 2514**			
ε2/ε2	3.06	(-1.61, 7.72)		ε2/ε2	3.67	(-1.76, 9.10)	
ε2/ε3	-0.85	(-2.14, 0.43)		ε2/ε3	0.08	(-1.40, 1.56)	
ε2/ε4	0.69	(-1.96, 3.34)		ε2/ε4	-0.77	(-3.84, 2.30)	
ε3/ε3	-	-		ε3/ε3	-	-	
ε3/ε4	0.39	(-0.69, 1.48)		ε3/ε4	-1.49	(-2.64, -0.35)	
ε4/ε4	-1.45	(-4.49, 1.59)	0.34	ε4/ε4	2.47	(-0.70, 5.64)	0.03
Key Stage 3							
**English**							
**N = 2337**				**N = 2150**			
ε2/ε2	3.78	(-3.44, 10.99)		ε2/ε2	2.75	(-4.68, 10.18)	
ε2/ε3	-0.20	(-2.26, 1.86)		ε2/ε3	1.46	(-0.71, 3.63)	
ε2/ε4	0.43	(-3.84, 4.70)		ε2/ε4	1.17	(-3.31, 5.64)	
ε3/ε3	-	-		ε3/ε3	-	-	
ε3/ε4	1.01	(-0.75, 2.78)		ε3/ε4	-0.93	(-2.63, 0.76)	
ε4/ε4	-2.27	(-6.95, 2.41)	0.61	ε4/ε4	3.41	(-1.36, 8.19)	0.26
**Maths**							
**N = 2419**				**N = 2188**			
ε2/ε2	1.52	(-7.38, 10.41)		ε2/ε2	1.11	(-8.30, 10.52)	
ε2/ε3	-1.39	(-3.89, 1.12)		ε2/ε3	-1.32	(-4.11, 1.48)	

ε2/ε4	1.21	(-3.97,	6.40)		ε2/ε4	-3.88	(-9.64,	1.87)	
ε3/ε3	-	-			ε3/ε3	-	-		
ε3/ε4	0.99	(-1.16,	3.14)		ε3/ε4	-0.60	(-2.79,	1.58)	
ε4/ε4	-4.62	(-10.24,	1.01)	0.34	ε4/ε4	5.19	(-0.83,	11.22)	0.31
**Science**									
**N = 2439**					**N = 2196**				
ε2/ε2	-1.99	(-11.74,	7.75)		ε2/ε2	-3.79	(-14.46,	6.88)	
ε2/ε3	2.31	(-0.43,	5.05)		ε2/ε3	-0.72	(-3.88,	2.44)	
ε2/ε4	1.57	(-4.07,	7.20)		ε2/ε4	0.01	(-6.46,	6.49)	
ε3/ε3	-	-			ε3/ε3	-	-		
ε3/ε4	1.36	(-0.99,	3.70)		ε3/ε4	1.26	(-1.21,	3.73)	
ε4/ε4	1.11	(-5.05,	7.27)	0.59	ε4/ε4	5.07	(-1.82,	11.97)	0.55

[a] Coefficient from linear regression, adjusted for age child’s age in months with ε3/ε3 genotype as reference group

[b] P values for heterogeneity from analysis of covariance model
